# Predicting coral-reef futures from El Niño and Pacific Decadal Oscillation events

**DOI:** 10.1038/s41598-020-64411-8

**Published:** 2020-05-08

**Authors:** Peter Houk, Anthony Yalon, Selino Maxin, Christy Starsinic, Andrew McInnis, Marine Gouezo, Yimnang Golbuu, Robert van Woesik

**Affiliations:** 1 University of Guam Marine Laboratory, UOG Station, Mangilao, GU 96923 USA; 2 Yap Community Action Program, Colonia, Yap FM. 96943 Federated States of Micronesia; 3 Conservation Society of Pohnpei, Kolonia, Pohnpei FM. 96941 Federated States of Micronesia; 4 Palau International Coral Reef Center, Koror, Palau 96940 Republic of Palau; 5 Florida Institute of Technology, 150W University Blvd, Melbourne, FL 32901 USA

**Keywords:** Climate-change ecology, Ecosystem ecology

## Abstract

El Niño Southern Oscillation (ENSO) events modulate oceanographic processes that control temperature and productivity in tropical waters, yet potential interactions with low frequency climate variability, such as the Pacific Decadal Oscillation (PDO), are poorly understood. We show that ENSO and PDO together predicted (i) maximum sea-surface temperatures (SST), which were associated with coral bleaching and declines in coral cover, and (ii) maximum chlorophyll-a concentrations, which were associated with high densities of coral-predatory *Acanthaster* starfish, across the tropical north Pacific Ocean since 1980. Asynchrony between the positive PDO and negative ENSO (i.e., La Niña) was associated with peaks in annual SST. By contrast, synchrony between the positive PDO and positive ENSO (i.e., El Niño) was associated with peaks in chlorophyll-a. Both conditions led to ecological disturbances and significant loss of coral cover, however, spatial models revealed where impacts to reefs were expected under varying climate scenarios.  The 2015/17 ENSO event was coupled with a positive PDO and resulted in high SST and *Acanthaster* abundances in eastern Micronesia, while positive coral growth occurred in western Micronesia.  Our novel approach for forecasting coral growth into the future may be applicable to other oceanic regions with differing oceanographic modulators.

## Introduction

Coral bleaching is a consequence of anomalous thermal-stress events that have led to  coral mortality in extreme conditions and to changes in the composition of remaining coral assemblages^1,2^. A recent increase in the frequency and intensity of thermal-stress events in the Pacific Ocean is attributed to the overprint of ocean warming on El Niño Southern Oscillation (ENSO)^2–4^. The overprint of warming in the central Pacific reduce the threshold and increase the likelihood of extreme ENSO events^5,6^. Yet, predicting where and when coral reefs are susceptible to ENSO events is challenging^2,7,8^. Consider for example the coral reefs in Micronesia that were exposed to strong ENSO events in 1997/98 and again in 2015/17. The 1997/98 ENSO was attributed to extensive coral bleaching in western Micronesia^9,10^, whereas the 2015/17 event impacted central and eastern Micronesia 2000 km farther eastward. These disturbance patterns suggest that our ability to predict reef futures relies on a better understanding of how ENSO combines with secondary, low frequency climate variability to regulate ocean temperatures across the tropical Pacific Ocean^11^.

Short-term predictive models can accurately forecast ocean temperatures 3–4 months into the future using autoregressive and linear inverse models that are extensions of recent climate observations^12^. These types of models, for example, predict thermal stress events and form the basis for coral bleaching alerts that are derived from satellite SST data^13–15^. By contrast, longer-term predictions that reveal the likelihood of future thermal anomalies are based on past temperature records from geological coral cores and on historical patterns of past temperatures (Thompson and van Woesik 2009). Representative concentration pathway (RCP) models, for example, predict SST trajectories over the next 50 to 100 years under varying CO_2_ emissions scenarios^8,16^. RCP models provide novel insight into patterns and processes associated with SST variation and rely upon smoothed annual trends over low-resolution geographic projections. The present study differs from both approaches and sought to predict changes in ocean temperatures across years to decades that were driven by climate oscillations associated with ENSO and the Pacific Decadal Oscillation (PDO).

While bleaching events are commonly associated with ENSO cycles^1,2^, the PDO represents a low frequency climate variability that is associated with changes in SST and productivity along a north-to-south gradient in the tropical and temperate Pacific Ocean. The north-to-south climatic shifts are associated with cool and warm PDO phases that can augment or diminish the influence of ENSO^17,18^. Although the atmospheric and oceanic processes driving the PDO are poorly understood, at least compared with ENSO, the present study relies upon the PDO indices as a measure of predictable oceanographic change as the science surrounding the PDO and other interdecadal oscillations continue to evolve^19^.

In addition to thermal stressors, coral reefs in the Pacific and Indian Oceans are subjected to repeated outbreaks of the coral-eating seastar *Acanthaster*. Several studies have demonstrated that the density of these coral predators are proportional to the extent of coral mortality, and that *Acanthaster* frequently spread across reefs like fires spread through forests^20,21^. These episodes of intensive coral predation have been linked to high nutrient concentrations for brachiolaria larvae of *Acanthaster*^22^. Such high nutrient concentrations are associated with river discharge^23,24^ or anomalous oceanographic conditions generating high coastal productivity, frequently detected as high chlorophyll-a concentrations^25,26^. Enhanced primary productivity can occur during ENSO events in some coral-reef localities that are otherwise nutrient depauperate^25^. For example, ENSO can cause dynamic shifts in coastal upwelling, currents, and regional rainfall^26^.

**Figure 1:**
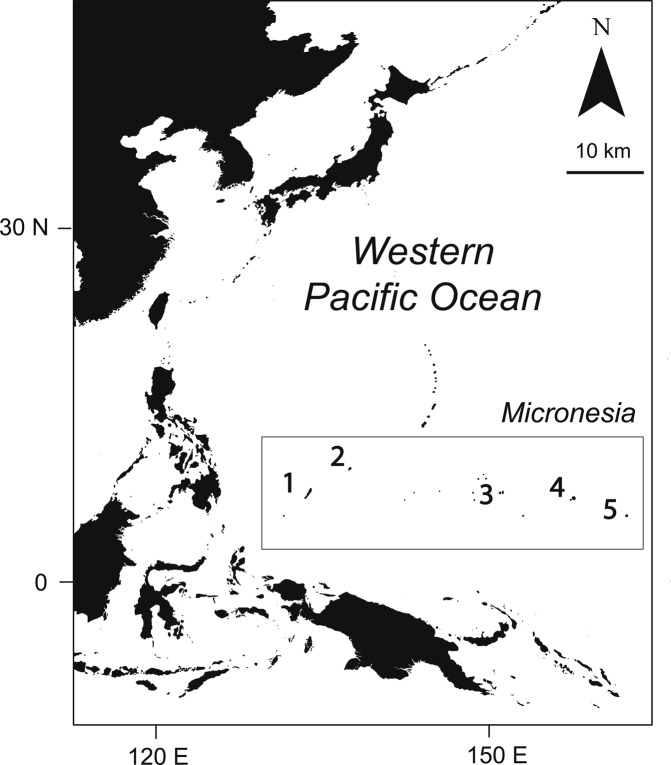
Map of the western Pacific Ocean with a geographical box surrounding the tropical North Pacific study region of Micronesia. Numbers indicate islands where long-term biological data were collected: (1) Palau, (2) Yap, (3) Chuuk, (4) Pohnpei, and (5) Kosrae. Map was created by author PH using ArcGIS software 10.2.2 and freely available base maps associated with the online account.

Here, we modelled the relationships between indices of climate modulators, thermal stress and chlorophyll-a concentrations, and the resultant change in coral cover across the tropical North Pacific (1° to 11°N, 130° to 165°E, Fig. 1). Our study revealed a strong interaction between these indices and changes to the coral reefs of Micronesia, demonstrating how ENSO cycles influence both heat transfer and productivity in a predictable, but spatially inconsistent manner.

## Materials and Methods

**Figure 2:**
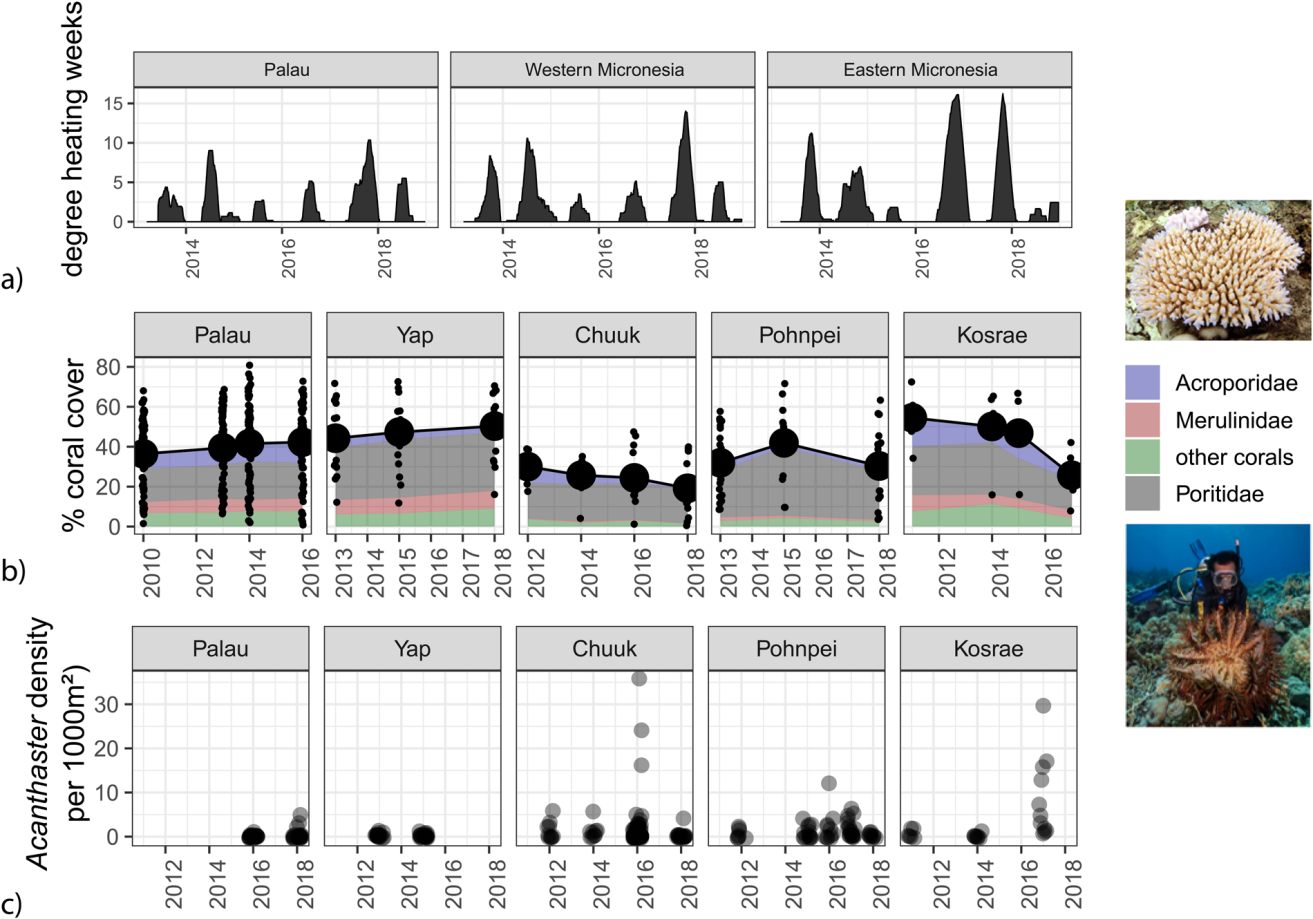
Oceanographic and biological data across the Micronesia study region. Degree heating weeks (**a**) provide an indication of heat stress accumulated in each sub-region since 2013, and highlight spatial differences associated with the 2015–2017 ENSO. Biological data show the dynamics of coral cover on each island (**b**) and the densities of *Acanthaster* cf. *solaris* observed at each site (**c**). Small black circles indicate coral cover at each site, whereas large black circles indicate island means. Color area fills provide a breakdown of the total coral cover into three major families, Acroporidae, Merulinidae, and Poritidae, and all other corals grouped together. Small black circles for *Acanthaster* densities represent numbers per site, or per 1000 m^2^. Photographs were taken by Simon Lorenz who provided written permission for use.

The present study was conducted in Micronesia, tropical northwestern Pacific Ocean (1° to 11°N, 130° to 165°E) (Fig. 1). Micronesia has a long history of disturbances impacting coral reefs. Widespread thermal-stress related coral bleaching was first documented in Palau, western Micronesia, following the 1998 ENSO^9^. Bleaching was also observed in Palau following the 2010 ENSO but not observed in eastern Micronesia^27^. By contrast, the recent ENSO event (2015–2017) showed greatest thermal stress in eastern Micronesia, particularly in Kosrae (Fig. 2).

The first reports of large populations of predatory sea stars (i.e., *Acanthaster* cf. *solaris*, or Crown-of-Thorns starfish, previously known as *Acanthaster planci*) occurred in Micronesia in the late 1960s and early 1970s. These events appeared to be novel, and spanned across Micronesia and the Pacific Ocean^28,29^. *Acanthaster* outbreaks have now been reported in all of the islands of Micronesia over the past three decades^21,25,30,31^, but the sizes of the recent populations have been consistently smaller than in the late 1960s. In the last decade, *Acanthaster* outbreaks have been most prevalent in Pohnpei (2009 and present study), Chuuk (2008, 2012, and present study), and Kosrae (present study), with fewer starfish found on the reefs of western Micronesia.

### Study design and datasets

We characterized coral bleaching and *Acanthaster* outbreak events across Micronesia from 2010 to 2017 by examining patterns between standardized biological data and satellite-derived oceanographic data. Biological data were collected as part of the long-term monitoring program in Micronesia that has been described in detail elsewhere^31^. Briefly, between 10 to 25 sites were established and monitored on each of the main islands of Micronesia. The number of sites was proportional to the size of the island and the amount of coral-reef habitat. A total of 82 sites were examined in the present study. Sites were selected to be representative of each island and were spread across differing reef types, wave exposures, and management regimes. During each survey, standardized methods were used to estimate the coverage of the main benthic substrates and to estimate the densities of *Acanthaster*. Five, 50 m transects were laid at the 5–8 m depth and a 0.5 × 0.5 m photograph was taken of the substrate at each 1 m interval. Within each photo, the benthic substrate was evaluated under five randomly placed points. Coral cover was aggregated into 4 taxonomic groups: Acroporidae, Merulinidae, and Poritidae, and all ‘other’ corals grouped together. These methods were selected to yield high statistical power to detect 30% relative change in dominant taxa^32^. Taxonomic coral groupings were selected based upon differences in coral growth rates, framework and habitat complexity, coral skeletal density, and sensitivity to disturbances^33,34^. Macroinvertebrate densities were counted within 4-m wide belt transects along each 50-m transect. The present study used counts of *Acanthaster* aggregated at the site-level (i.e., density per 1000 m^2^).

A suite of satellite-derived oceanographic data were collected from the National Oceanic and Atmospheric Administration (NOAA) ERDDAP server (https://coastwatch.pfeg.noaa.gov/erddap/index.html). Sea surface temperature (SST) data were derived from the 1° HadiSST dataset between 1980 and the present, using this timeframe to ensure the most consistent data source and data quality. Chlorophyll-a concentrations data were derived from the European Space Agency Ocean Color Climate Change Initiative dataset version 3.1 that contained merged MERIS, Aqua-MODIS, SeawWIFS, and VIIRS data. In addition, 5-km degree-heating-week data were collected from the NOAA Coral Reef Watch program to augment SST data and provide another indication of heat stress (https://coralreefwatch.noaa.gov/satellite/dhw.php).

### Data analyses

Zero-inflated hurdle models were used to assess *Acanthaster* densities through time^35^. Zero-inflated models had two parts: (i) a binomial-model part that estimated the probability of finding *Acanthaster* at any given site in any given year, and (ii) a second part that estimated densities. The hurdle model produces two terms and two P-values, one for *Acanthaster* presence and one for *Acanthaster* density. We found clear relationships during the recent 2015–2017 ENSO event between: (i) high chlorophyll-a concentrations and increases in *Acanthaster*, and (ii) high SST and *Acanthaster* and coral cover decline. We next sought to understand how the major oceanographic modulator, El Niño Southern Oscillation (ENSO), may be influenced by the state of the Pacific Decadal Oscillation (PDO) to predict SST and chlorophyll-a concentrations through time.

Standard ENSO and PDO indices (https://www.esrl.noaa.gov/psd/enso/mei/) (http://research.jisao.washington.edu/pdo/) were downloaded to examine their ability to predict trends in SST and chlorophyll-a concentrations from 1980 to 2018 and 1998 to 2018, respectively, across our study region in Micronesia (Fig. 1). SST and chlorophyll-a concentrations were first averaged for all pixels in our geographic study region during each month for the years 2010 to 2017. We then calculated the annual and summertime monthly mean for SST and the annual and wintertime monthly mean for chlorophyll-a concentrations. This process focused on the seasons when SST and chlorophyll-a were highest. Least squares regressions were then used to assess the predictive power of four terms: ENSO, PDO, ENSO x PDO, and a sequential dummy variable for time. ENSO and PDO were only moderately correlated suggesting that they have separate contributions to the modeling process despite their known positive correlation (r = 0.58 and 0.68, Pearson’s correlations between ENSO and PDO indices when examining monthly values or annual averages, respectively). Best-fit models were assessed by their R^2^, P-values, and their Akaike Information Criterion (AIC) scores, and projected as spatial probability maps for all individual pixels (1°) across Micronesia. Final maps were smoothed by averaging across the eight surrounding pixels. All spatial regressions and mapping were conducted in R using packages raster, ncdf4, and rgdal^36–39^.

We next tested whether the same analytical framework could predict the rate of change in coral cover across our study region. Generalized linear mixed models tested whether ENSO, PDO, and ENSO × PDO interactions could predict site-based changes in coral cover, calculated as the percentage differences per year^40^. Because biological surveys were occasionally conducted before the summer and winter periods that were associated with oceanic indices, we also used ENSO and PDO as predictors during the year prior to field surveys.. For this process we assigned the following variables as random effects in our generalized linear mixed model: (1) island, (2) reef type, and (3) individual site. It would have been desirable to also include the abundance of *Acanthaster* densities as a predictor variable in the model, but the abundance estimates were not available for all years when coral data were collected. Regardless, we show that high *Acanthaster* densities were present across the same 2015 to 2017 study period associated with high SSTs and coral bleaching. Therefore, a change in coral cover was attributed to both thermal stress and predation pressure. We first built a null model, and then compared subsequent models with the null model using analyses of variance (ANOVA) to test between the residual deviance estimates. The best-fit model was selected based upon this stepwise comparison process and the AIC scores.

## Results

**Figure 3:**
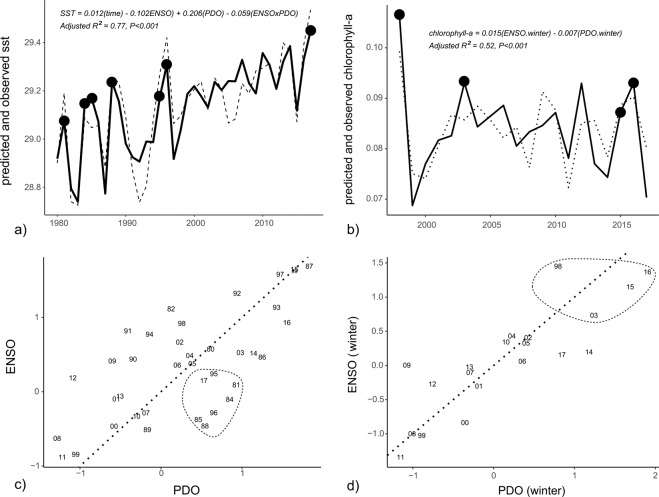
Best-fit regression models showing the predicted (dashed) and observed (solid) SST (**a**) and chlorophyll-a (**b**) concentrations associated with the geographical study region. Correlation plots for ENSO and PDO model terms are provided to highlight criteria associated with SST (**c**) and chlorophyll-a (**d**) maximum. Data points enclosed by the dotted circles in the correlation plots (**c,d**) correspond to the large black circles in the SST and chlorophyll-a trajectories (**a,b**).

The 2015–2017 ENSO was associated with major declines in coral cover and significant increases in *Acanthaster* densities, although there were spatial differences across the study region (Fig. 2). The negative phase of the ENSO (i.e., La Niña) was associated with the highest annual SST in 2016 and 2017 and caused significant coral loss on reefs in eastern Micronesia (Figs. 2 & 3a). Coral cover declined by 20 – 50% between 2010 to 2017 in eastern Micronesia, where degree heating weeks were highest. *Acanthaster* densities were highest between 2015 and 2017 for Pohnpei, Chuuk, and Kosrae, when ENSO phases switched from positive to negative (i.e., El Niño to La Niña) (P < 0.05, hurdle models comparing both presence and abundance across years, Fig. 2). Acroporid corals were most vulnerable to both thermal stresses and high *Acanthaster* densities (Fig. 2). By contrast, the western Micronesia reefs of Palau and Yap fared best through this period, with coral cover increasing from 2010 to 2017.

**Figure 4:**
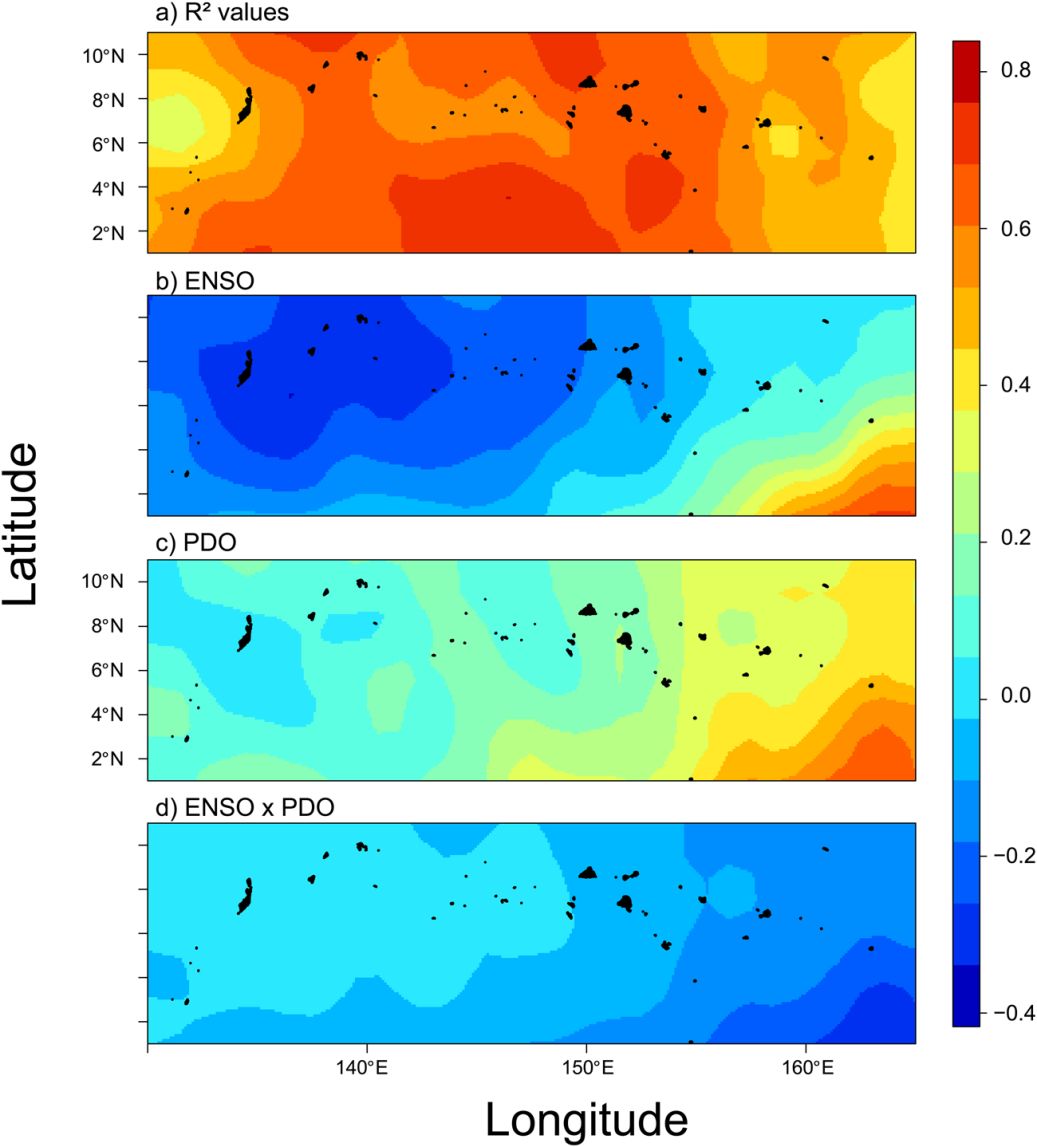
Smoothed spatial regressions highlighting the variation in R^2^ fit values (**a**) and model estimates for each term (**b**-ENSO, **c**-PDO, and **d**-ENSO × PDO). Blue colors indicate negative changes in SST with indices, whereas yellow and red colors indicate positive changes in SST with indices. Note the same scale is used for R^2^ values and model estimates. Maps were created by author PH using the R computing platform and packages raster, ncdf4, and rgdal^38–41^.

**Figure 5:**
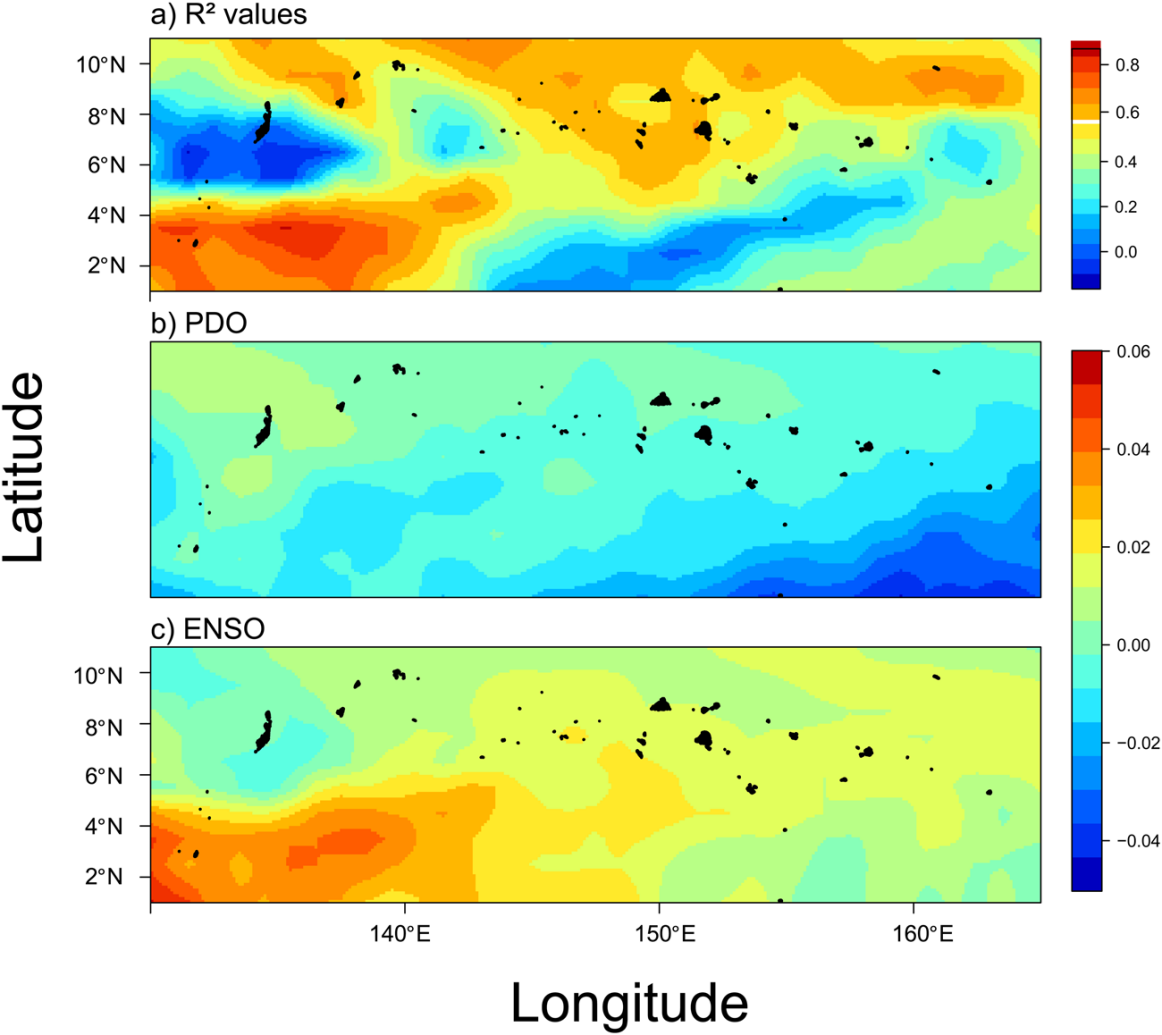
Smoothed spatial regressions highlighting the variation in R^2^ fit values (**a**) and model estimates for each term (**b**-PDO-winter, **c**-ENSO-winter). Blue colors indicate negative changes in chlorophyll-a with indices, while yellow and red colors indicate positive changes in chlorophyll-a with indices. Note the different scales used for R^2^ values and model estimates. Maps were created by author PH using the R computing platform and packages raster, ncdf4, and rgdal^38–41^.

We next examined whether ENSO and PDO predicted SST and chlorophyll-a concentrations that were driving the trends in coral cover. Regionally-averaged SST and chlorophyll-a concentrations were both accurately predicted from ENSO and PDO indices, with 77% and 52% of the variance explained by each respective model (Fig. 3a,b). Interestingly, between 1980 and 2017, negative ENSO coupled with positive PDO indices were correlated with peaks in SST (Fig. 3a,c). Spatially, SST was best predicted in central Micronesia where highest R^2^ values existed, while weaker predictions existed in eastern and western Micronesia (Fig. 4). By contrast, when ENSO and PDO indices were both positive, regional chlorophyll-a concentrations were highest (Fig. 3b,d). Chlorophyll-a data were available since 1998 limiting hindcasting to twenty years (1998 to 2017). Positive ENSO increased chlorophyll-a in southwestern Micronesia below 4°N, whereas negative PDO increased chlorophyll-a in southeastern Micronesia, especially in our easternmost study island of Kosrae (Fig. 5). In response, the highest densities of *Acanthaster* were found in eastern Micronesia with peak chlorophyll-a concentrations between 2015 and 2017 (Figs. 2 & 3b). However, the initiation of large *Acanthaster* outbreaks and localized culling programs in Kosrae, eastern Micronesia, coincided with the negative PDO prior to 2014 based upon local observations and photographs.

Mixed regression models revealed that ENSO and PDO together predicted the changes in percentage coral cover when allowing for random variation among islands (χ^2^ = 11.7, P < 0.01 comparing best-fit model with the null model, Supplementary Fig. S1). Positive ENSO, (i.e., El Niño), increased coral cover and was associated with cooler waters, whereas positive PDO decreased coral cover and was associated with warmer waters. Therefore, the greatest loss in coral cover occurred in 2016 and 2017 when ENSO was negative or even neutral, and PDO was positive. Yet, there were notable spatial differences. Eastern Micronesia was very sensitive to these conditions; however, western Micronesia was not impacted. Instead, western Micronesia was impacted by temperature stress when ENSO and PDO were both negative, as they were in 1997/98 when significant coral loss occurred.

## Discussion

ENSO events coupled with PDO variability accurately predicted changes on the coral reefs of Micronesia. These events influenced SST and chlorophyll-a concentrations, which led to coral bleaching and the regional emergence of *Acanthaster* coral predator populations during the recent 2015/17 ENSO. While ENSO events were primary drivers of SST and chlorophyll-a in the tropical North Pacific, the state of the PDO determined where they were highest. Positive PDO regimes indicated that warmer than usual SST existed in the western tropical Pacific. The presence of warmer waters shifted the migration of ENSO events by pushing cooler waters with higher chlorophyll-a concentrations to eastern Micronesia during positive ENSO (i.e., El Niño). As El Niño diminished and flipped to La Niña, these conditions appeared to limit the westward migration of warm waters along the equator. In sum, the positive PDO before and during the 2015/17 ENSO favored more *Acanthaster* outbreaks and greater regional heating in eastern Micronesia, where localized *Acanthaster* outbreaks were first observed prior to 2014.

More broadly, this study demonstrated  how integrating ENSO and PDO indices into forecasting could improve predictions of where local disturbances across the tropical North Pacific would be expected under any user-defined climate scenarios ^18,41^. While many studies have revealed that climate-induced bleaching is increasing in frequency^2^, especially at latitudes 15 to 20 degrees from the Equator over the last decade^42^, the enhanced spatial resolution of the present study suggested that ENSO cycles should not be simply equated with return periods of thermal stress. Instead, combined forecasting and hindcasting using secondary indicators of low frequency oceanic variability, such as the PDO, can improve predictions. We recognize the ongoing debate surrounding interdecadal climate oscillations and the lack of clarity surrounding underlying processes driving interdecadal variability^19^. We do not advocate for or against a process causing interdecadal variability in the tropical Pacific, but instead relied upon current indicators of ENSO and PDO as model inputs to examine coupling between oceanic variability and ecological change.

Extending our ability to predict disturbance events months to years in advance may benefit regional and local management efforts. Yet, whether managing local stressors might mitigate the impacts of climate change has been debated^43–45^. Some studies suggest that climate change is too powerful of a force and any gains from localized management are offset by repeat disturbances of thermal stress through time^44,46^. Alternatively, other studies suggest that local management can buy time and make local reefs temporally more resilient^45,47^. Our results help to reconcile this debate by balancing predictions of coral loss associated with climate variability against predictions of coral gains expected from local management. These types of analyses might highlight localities where local management can produce noticeable returns despite expected losses from interannual climate oscillations. Further, our study provided the foundation to forecast the impacts of climate change across numerous coral taxa, thereby improving our understanding of coral  growth, habitat complexity, and numerous processes on reefs that are associated with changing coral assemblages^48,49^.

We conclude that ENSO and PDO have been used to predict fisheries productivity in the north Pacific, wildfires in the southwest of the United States, and both localized droughts and global rainfall patterns^50–53^. These advances in prediction allow forest, wildlife, and fisheries managers a stronger basis for annual and longer-term decision-making policies that are needed to manage stocks. We provide the first insight into how PDO and ENSO cycles predicted SST, chlorophyll-a concentrations, and changes to coral cover across the tropical north Pacific Ocean. These results may be transferrable to other oceanic regions to help predict coral reef status at even larger spatial scales.

## Supplementary information

Supplementary information.
