# Supplementary material for: Predicting coral-reef futures from El Niño and Pacific Decadal Oscillation events
Source: Sci Rep. 2020 May 8;10:7735. doi: 10.1038/s41598-020-64411-8 (PMC7210262; doi:10.1038/s41598-020-64411-8)
Supplement: Supplementary file 1 — Supplementary information. [file 41598_2020_64411_MOESM1_ESM.docx]

**Supporting information**

# Predicting coral-reef futures from El Niño and Pacific Decadal Oscillation events

**Authors:** Peter Houk^*1^, Anthony Yalon^2^, Selino Maxin^3^, Christy Starsinic^1^, Andrew McInnis^1^, Marine Gouezo^4^, Yimnang Golbuu^4^, Robert van Woesik^5^

**Affiliations:** ^*1^University of Guam Marine Laboratory, UOG Station, Mangilao, GU. 96923; ^2^Yap Community Action Program, Colonia, Yap, FM. 96943; ^3^Conservation Society of Pohnpei, Kolonia, Pohnpei, FM. 96941; ^4^Palau International Coral Reef Center, Koror, Palau. 96940; ^5^Florida Institute of Technology, 150 W University Blvd, Melbourne, FL. 32901


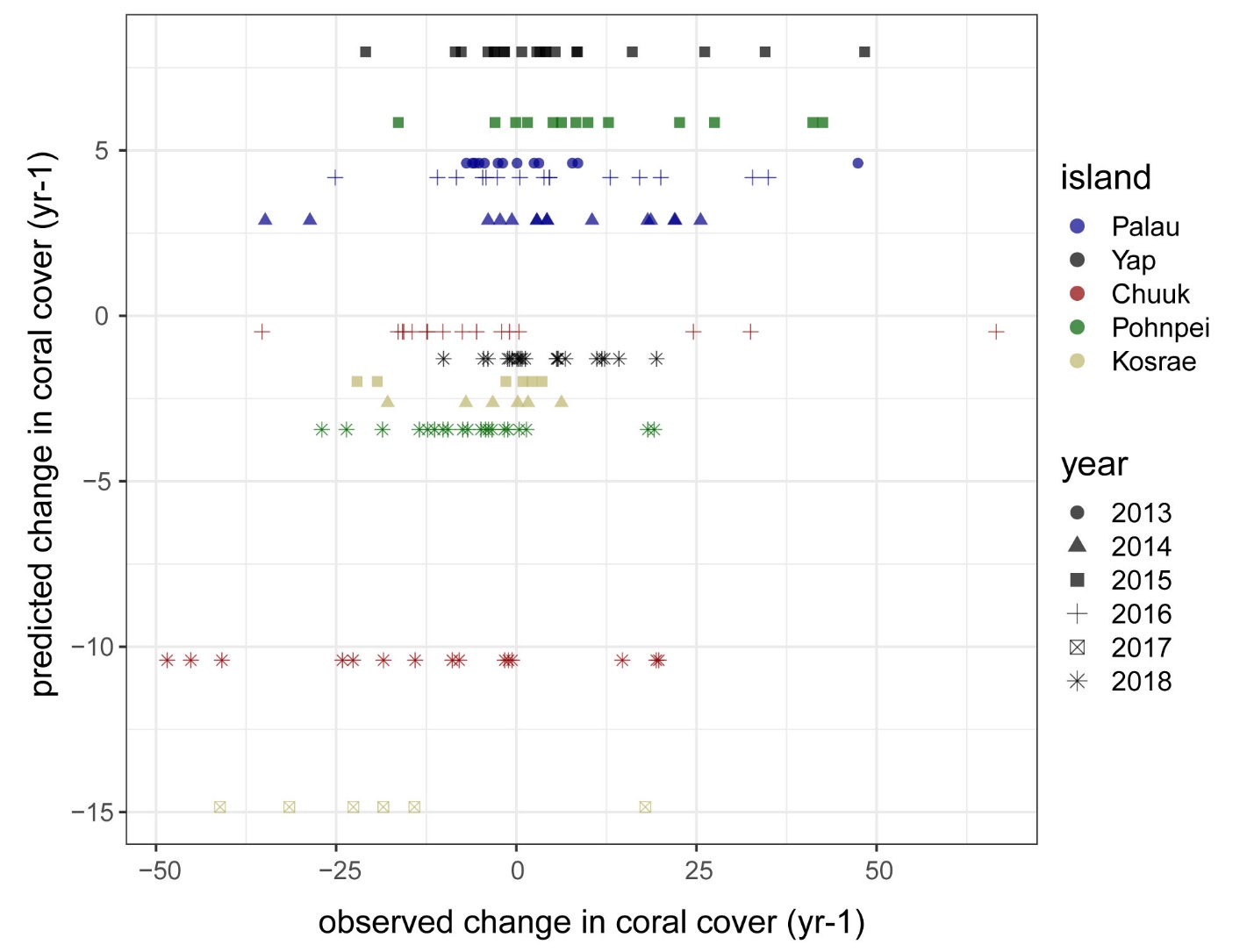


**Figure S1**. Predicted versus observed changes in coral cover from best-fit, mixed-effect regression modeling. Each symbol represents the percent change in coral cover at one island in one year. The PDO and ENSO oceanographic indices that were significant predictors of change differed annually, so multiple intercepts (i.e., lines) existed for each island depending upon the amount of biological data available.
